# Extreme temperatures modulate gene expression in the airway epithelium of the lungs in mice and asthma patients

**DOI:** 10.3389/fmed.2025.1531154

**Published:** 2025-04-17

**Authors:** Firdian Makrufardi, Syue-Wei Peng, Kian Fan Chung, Marc Chadeau-Hyam, Kang-Yun Lee, Ta-Chih Hsiao, Kin-Fai Ho, Desy Rusmawatiningtyas, Indah Kartika Murni, Eggi Arguni, Yuan-Hung Wang, Shu-Chuan Ho, Feng-Ming Yang, Kai-Jen Chuang, Sheng-Chieh Lin, Hsiao-Chi Chuang

**Affiliations:** ^1^International Ph.D. Program in Medicine, College of Medicine, Taipei Medical University, Taipei, Taiwan; ^2^Department of Child Health, Faculty of Medicine, Public Health, and Nursing, Universitas Gadjah Mada – Dr. Sardjito Hospital, Yogyakarta, Indonesia; ^3^School of Respiratory Therapy, College of Medicine, Taipei Medical University, Taipei, Taiwan; ^4^Imperial College London, National Heart and Lung Institute, London, United Kingdom; ^5^Department of Epidemiology and Biostatistics, School of Public Health, Imperial College London, London, United Kingdom; ^6^MRC Centre for Environment and Health Imperial College London, London, United Kingdom; ^7^Division of Pulmonary Medicine, Department of Internal Medicine, School of Medicine, College of Medicine, Taipei Medical University, Taipei, Taiwan; ^8^Division of Pulmonary Medicine, Department of Internal Medicine, Shuang Ho Hospital, Taipei Medical University, New Taipei City, Taiwan; ^9^Graduate Institute of Environmental Engineering, National Taiwan University, Taipei, Taiwan; ^10^JC School of Public Health and Primary Care, The Chinese University of Hong Kong, Hong Kong, China; ^11^Graduate Institute of Clinical Medicine, College of Medicine, Taipei Medical University, Taipei, Taiwan; ^12^Department of Medical Research, Shuang Ho Hospital, Taipei Medical University, New Taipei City, Taiwan; ^13^School of Public Health, College of Public Health, Taipei Medical University, Taipei, Taiwan; ^14^Department of Public Health, School of Medicine, College of Medicine, Taipei Medical University, Taipei, Taiwan; ^15^Department of Pediatrics, School of Medicine, College of Medicine, Taipei Medical University, Taipei, Taiwan; ^16^Division of Allergy, Asthma, and Immunology, Department of Pediatrics, Shuang Ho Hospital, Taipei Medical University, New Taipei City, Taiwan; ^17^Cell Physiology and Molecular Image Research Center, Wan Fang Hospital, Taipei Medical University, Taipei, Taiwan

**Keywords:** airway epithelium, climate change, extreme weather, lung, thermal

## Abstract

**Background:**

The objective of this study was to examine the effects of extreme temperatures on the gene signature and pathways of airway epithelial cells in mice and asthma patients.

**Methods:**

We investigated the effects of temperature exposure at normal (22°C), and extreme low (10°C), high (40°C) and temperature fluctuation (40°C for 2 h followed by 10°C for next 2 h) in B6.*Sftpc-CreER*^T2^*;Ai14(RCL-tdT)*-D mice and pediatric and adult patient’s airway epithelial exposed to extreme temperatures.

**Results:**

We observed that Mmp8, Sftpb, Cxcl15 and Cd14 were significantly upregulated in airway epithelial cells in mice model. Cma1, Kit, Fdx1, Elf1a, Cdkn2aipnl, Htatsf1, Mfsd13a, Gtf2h5, Tiam2, and Trmt10c were significantly upregulated in 40°C exposure in airway epithelial cells. Sftpc, Gpr171, Sic34a2, Cox14, Lamp3, Luc7l, Nxnl, Tmub2, Tob1, and Cd3e genes were significantly upregulated in 10°C exposure group. Pediatric asthma subjects in the extreme high temperature group consistently showed decreased Wfdc21, Cib3, and Sftpc, at the same time increased Tiam2 and Cma1 expression, while in the extreme low temperature group exhibited consistently higher expression of Sftpc and Nxnl, at the same time decreased Wfdc21, Cib3, Cma1, and Dld expression. Notably, the mice in the extreme temperature fluctuation group showed decreased Wfdc21, Cib3, Gpr171, and Cttnbp2 expression, while increased Hbb-bs expression. Adult asthma subjects in the extreme temperature fluctuation group showed consistently decreased Wfdc21, Cib3, Gpr171, and Cttnbp2 expression, while increased Tiam2 and Cma1 expression. We observed that the mild, moderate, and severe asthma subject in the extreme low temperature group showed increased Tob1, Mub2, Sic34a2, Sftpc, Nxnl, Luc71, Lamp3, Gpr171, Cox14, and Cd3e expression, while in the severe asthma subjects showed increased expression in all temperature exposure group.

**Conclusion:**

Our study highlights the effects of extreme temperatures on the gene signature of the airway epithelium in both mice and asthma patients. These findings suggest that extreme temperatures modulate gene expression in the airway epithelium, potentially serving as clinical indicators or biomarkers in response to climate change.

## 1 Introduction

Climate change has altered the frequency, intensity, and geographic distribution of extreme temperatures and abrupt temperature fluctuations, thereby increasing the risk of human illness and mortality (1). The Intergovernmental Panel on Climate Change (IPCC) assessment report confirmed that climate change poses a direct threat to respiratory health, including asthma (2). A previous study on asthma subjects found that extreme cold and extreme heat were associated with an increase in asthma severity and mortality (3). Another study in asthma patients with exposure to sudden temperature fluctuations found an association between the decrease in forced expiratory volume in 1 second (FEV_1_) and forced vital capacity (FVC) (4). Optimal temperature and relative humidity are necessary for critical metabolic processes and metabolic adaptations, including nutrient mobilization, partitioning, and mitochondrial energy production (5). However, extreme temperatures program cell death through the generation of reactive oxygen species (ROS) (6). Nonetheless, the mechanism by which these extreme temperatures cause lung impairment remains unclear.

The lungs, as primary organs exposed to extreme temperatures are particularly vulnerable during acute asthma exacerbations (7). A previous study observed that climate change, manifested in extreme temperatures, increased the risk of asthma events, asthma symptoms, and asthma-related emergency department visits (8). Airway epithelial cells serve as lung progenitors, playing a crucial role in the regeneration of optimal respiratory function following epithelial injury in climate change (9). High temperature caused bronchial epithelium thickness, subepithelial fibrosis, and inflammatory cell infiltration around airways, triggered IL-4, IL-1β, IL-6, and TNF-α release and shifted TH1/TH2 balance to TH2 (10). Another study also observed increased mitochondrial superoxide generation and mitochondrial DNA damage in airway epithelial cells of asthma patients, suggesting the role of oxidative stress in airway epithelial cells contributed to asthma (11). Together, these suggested that extreme temperature events could result in lung injury through disruption of airway epithelium function.

Few studies have investigated the impact of climate change on extreme temperature effects in airway epithelial cells of the lung, and the potential regulatory role of climate change in temperature-induced lung alterations remains unclear. We investigated the effects of extreme temperatures on the gene signature and pathways of airway epithelial cells in mice and asthma patients. Understanding the gene signature associated with extreme temperature events is essential for elucidating the regenerative processes of airway epithelial cells in response to such conditions under the influence of climate change.

## 2 Materials and methods

### 2.1 Animals

The mouse experiments were conducted in accordance with the guidelines of the Taipei Medical University Animal and Ethics Review Committee of the Laboratory Animal Center (Taipei, Taiwan; IACUC: LAC-2022-0317). Both male and female 7 weeks-old B6.*Sftpc-CreER*^T2^*;Ai14(RCL-tdT)*-D mice (Biolasco, Taipei, Taiwan) were used in this study, with equal numbers of each sex to ensure balanced representation. The temperature of the environment was 22 ± 2°C with relative humidity set at 55% ± 10%. The mice were housed in the laboratory animal center of Taipei Medical University with 12:12 h light: dark cycle, provided with Lab Diet 5001 (PMI Nutrition International, United States), and allowed access to water ad libitum.

### 2.2 Temperature exposure in mouse

**FIGURE 1:**
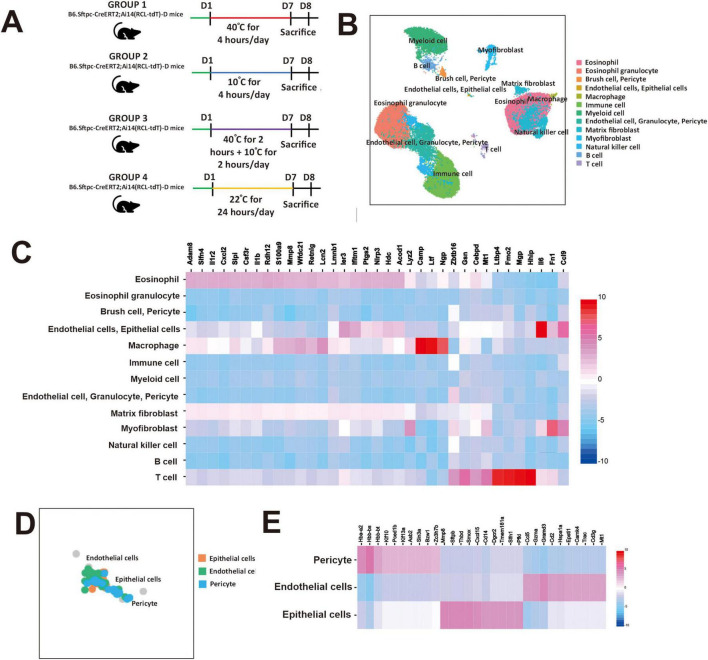
Gene expression in mouse lung cells across 13 clusters in response to extreme temperature. **(A)** Schematic of experimental design under extreme temperature conditions. **(B)** Uniform manifold approximation and projection (UMAP) of lung cells from mice are color-coded by cell type. **(C)** Heat map of the relative average expression of the most strongly enriched genes for each cluster [log(fold change) of one cluster versus all others], grouped by cell type. All gene expression values are normalized across rows. **(D)** UMAP visualization of lung cells from mice, identified through graph-based clustering, is indicated by color and annotated as epithelial cells, endothelial cells, and pericytes. **(E)** Heat map of the average expression of the most strongly enriched genes for the epithelial cluster [log(fold change)]. All gene expression values are normalized across rows.

The B6.*Sftpc-CreER*^T2^*;Ai14(RCL-tdT)*-D mice were exposed to normal (22°C), and extreme low (10°C), high temperature (40°C), and temperature fluctuation (40°C for 2 h then 10°C for next 2 h), with three mice in each group, under normal relative humidity (65%) (Figure 1A). The exposure was 4 h/day for 7 days. Details and illustrations of the advanced thermal whole-body exposure system are described in the Supplementary Figure 1. The inlet air was filtered through a HEPA filter to remove particulate pollutants and through charcoal and/or a denuder to eliminate gaseous pollutants and organic compounds prior to temperature and relative humidity control. The exposure system was equipped with a thermal meter and a humidity monitor to monitor the exposure conditions. Finally, mice were euthanized by injecting 100 μL of zoletil-rompun solution (100 mg/kg zoletil and 10 mg/kg rompun) (12).

### 2.3 Single cell RNA sequencing (scRNA-seq) analysis

We perfused the lungs by injecting 10 mL of sterile cold PBS into the right ventricle of the heart to eliminate red blood cells (12). A whole lung sample, including the right and left lungs, was collected from each mouse at normal (22°C), extreme low (10°C), high (40°C), and temperature fluctuation (40°C–10°C). Lung dissociation was performed and the samples were analyzed using the GemCode Single Cell Platform using the Chromium Fixed RNA Kit (10X Genomics, Pleasanton) following the protocol provided by the company. The Cell Ranger software pipeline (version 2.0) from 10X Genomics was used to analyze the scRNA-Seq data. The Seurat R package (version 1.4.0.14^1^) was utilized to process the UMI count matrix and to eliminate potential multiple captures (13). Cell prefiltering was conducted based on quality control standards, encompassing the expression of 2000–8000 genes and the total UMIs amounting to less than 20,000. Automatic labeling of cell types within clusters derived from single-cell RNA sequencing data was performed using the scCATCH (14). Loupe™ Cell Browser v2.1 (10X Genomics) was used to analyze gene expression in airway epithelial *Epcam* positive (Epcam^+^) cells. Details of the scRNA-Seq analysis is described in the Supplementary materials.

### 2.4 Gene expression datasets in airway epithelium of pediatric and adult asthma patients

We selected gene expression datasets from the Gene Expression Omnibus (GEO^2^) database of the National Center for Biotechnology Information. We included both pediatric and adult asthma patients to capture age-specific differences in airway epithelium biology. The datasets are further described in the Supplementary Table 1. The dataset criteria were established as follows: (a) the dataset must include both healthy controls and asthmatic patients, and (b) the samples must be derived from airway epithelial brushings. Transcriptomic data from normal and asthmatic patients who underwent bronchoscopy to obtain bronchial brushings were included in the analysis from public gene expression datasets. Gene expression data were gathered from 23 healthy controls and 23 patients with asthma in pediatric populations. The pediatric age groups in this study were defined according to the guidelines of the American Academy of Pediatrics (15). Additionally, data from epithelial brushings were collected from a diverse group, including 10 healthy controls, 10 patients with mild asthma, 10 patients with moderate asthma, and eight patients with severe asthma among adult participants. We further identified the top 10 highly expressed genes from single-cell RNA sequencing of airway epithelial Epcam-positive (Epcam^+^) cells in mice, and subsequently examined their expression in pediatric and adult gene expression datasets (16).

### 2.5 Temperature assessment of pediatric and adult asthma patients

This research concentrated on weather data from selected stations in the United States to effectively study the extreme temperature in each state. Weather data were collected from 44 weather stations via accessible online databases^3^ maintained by the National Oceanic and Atmospheric Administration (NOAA). All available temperature and precipitation data were collected for each weather station, including daily maximum, minimum, and average temperature data. Stations containing data dating back a century or more were chosen as the optimal stations for analysis. The temperature for each case was estimated using the data from the weather station at the coordinate points of each data point at the time of admission. Patients exposed to extreme temperatures in the study included those exposed to extreme heat [defined as the 90th percentile or above in a single day (40.2°C in average)], extreme cold [defined as the 10th percentile or below in a single day (9.8°C in average)], and extreme temperature fluctuation [intra-day temperature variability (25.3°C in average)] were classified into extreme low (corresponding to 10°C in mice), high (corresponding to 40°C in mice), and temperature fluctuation (corresponding to 40°C–10°C in mice) groups, respectively, and linked to our mice model. This analysis was performed to correlate the environmental temperature extremes experienced by pediatric and adult asthma patients with the temperature exposure in our mouse model. The characteristics and details of the temperature exposure of the study subjects are shown in the Supplementary Table 2 and Supplementary Figure 2.

### 2.6 Differential expression and enrichment analyses

The differential expression of DEGs was visualized as volcano plots by the “ggplot2” package in R. DEGs were identified based on the following criteria: | Log2 (fold change) FC | > 0.5 and *P*-value < 0.05. To identify the regulatory mechanisms associated with hub genes, we used the R package “clusterProfiler” to carry out enrichment analyses (17). The screening criterion for Biological Process (BP) or Kyoto Encyclopedia of Genes and Genomes (KEGG) terms was an adjusted *P*-value of less than 0.05.

### 2.7 Protein-protein interaction (PPI) and gene-gene interaction (GGI)

A PPI network was built to display the interaction between protein targets in the crucial modules by the STRING database (version 11.0^4^). In this study, we investigated the top 10 highly expressed genes in the single-cell RNA sequencing from mice to the asthma patients. We set the minimum required interaction score to 0.4 (medium confidence) and visualized them using the Cytoscape software (version 3.8.2). In addition, we used the GeneMANIA database^5^ to construct gene-gene interaction (GGI) networks as well as co-expression and enrichment pathways interacting with hub genes. GeneMANIA database can further demonstrate the interactions between these hub targets, such as co-expression, co-localization, pathways, predicted physical interactions, shared protein domains and genetic interactions. Each interaction was shown in a different color.

## 3 Results

### 3.1 Gene expression in mice lungs in response to extreme temperatures

We classified mouse cells in the all temperature groups into 16 groups of cell types, which included 13 clusters of identified multiple cells (eosinophil, eosinophil granulocyte, brush cell, pericyte, endothelial cells, epithelial cells, macrophage, immune cell, myeloid cell, granulocyte, pericyte, matrix fibroblast, myofibroblast, natural killer cell, B cell, and T cell) (Figure 1B). Next, we observed that *Adam8*, *Sfln4*, *Ilir2*, *Cxcl2*, *Slpl*, *Csfr3r*, *Il1b*, *Rdh12* and *S100a9* expression were significantly increased in eosinophil cluster (Figure 1C). In addition, *Ler3*, *Ifitm1*, *Ptgs2*, *Nlrp3*, *Hdc*, *Acod1*, *Il6*, *Fn1*, and *Ccl9* expression were significantly increased in the endothelial and epithelial clusters. We also observed that *Zbtb16*, *Gsn*, *Cebpd*, *Mt1*, *Ltbp4*, *Fmo2*, *Mgp*, and *Hhip* were significantly increased in T cell cluster. We then selected the fourth cluster and investigated epithelial cells, endothelial cells, and pericytes (Figure 1D). This cluster was chosen because it contained a substantial proportion of epithelial cells, which are the primary barrier and immune-responsive cells in the airway and play a crucial role in asthma pathophysiology (9). Furthermore, this cluster was selected to observe airway epithelial gene signatures in comparison to surrounding stromal cells under exposure to extreme temperature conditions. We observed that *Mmp8*, *Sftpb*, *Thbd*, *Smox*, *Cxcl15*, *Cd14*, *Dgcr2*, *Tmem181a*, *Slfn1*, and *Pfkl* were significantly increased in epithelial cells, whereas significantly decreased in endothelial cells and pericytes (Figure 1E).

### 3.2 Extreme temperatures effect on gene expression in mice airway epithelial cells

**FIGURE 2:**
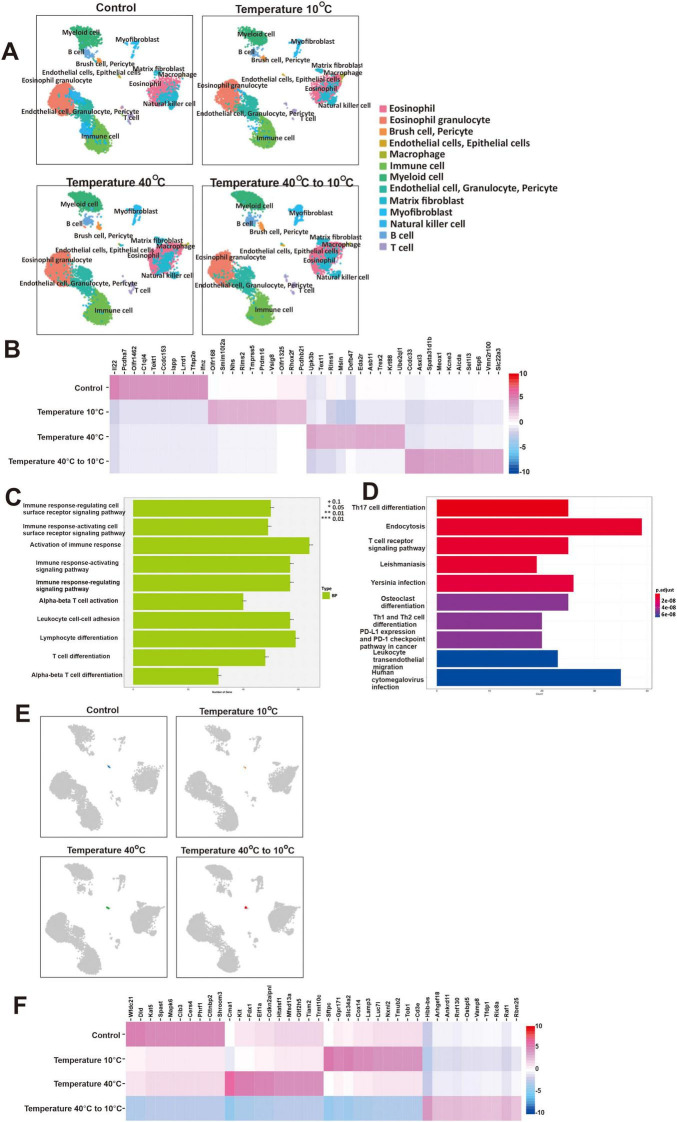
Extreme temperatures play a crucial role in modulating gene expression in mice airway epithelial cells. **(A)** The UMAP embedding illustrates the clustering of the 13 distinct cell types identified within mouse lungs, modulated by extreme temperatures (40°C and 10°C) and temperature fluctuations (40°C–10°C). **(B)** The heat map of the relative average expression of the most strongly enriched genes for each group [log(fold change) of one group versus all others], grouped by temperature exposure. All gene expression values are normalized across rows. **(C)** Gene ontology enrichment analysis related to significantly enriched biological processes (BP) based on the top differentially expressed genes in temperature fluctuations (40°C to 10°C) compared to control. **(D)** Kyoto Encyclopedia of Genes and Genomes (KEGG) enrichment pathway in temperature fluctuations (40°C–10°C) compared to control. **(E)** The UMAP embedding illustrates the clustering of airway epithelial cells identified within mouse lungs, influenced by both extreme temperatures (40°C and 10°C) and temperature fluctuations (40°C–10°C). **(F)** The heat map illustrates the relative average expression levels of the most significantly enriched genes in airway epithelial cells for each group, showing the [log(fold change) of one group versus all others].

The identified clusters were further examined under extreme temperatures and temperature fluctuation exposures in mouse lung samples (Figure 2A). We observed that the *Olfr168*, *Smim10l2a*, *Nhs*, *Rims2*, *Tmprss5*, *Prdm16*, *Vsig8*, *Olfr1325*, *Rhox2f*, and *Pcdhb21* expression were the top 10 genes significantly upregulated in 10°C exposure group (Figure 2B). On the other hand, *Ccdc33*, *Asxl3*, *Spata31d1b*, *Meox1*, *Kcne3*, *Alcda*, *Sel1l3*, *Esp6*, *Vmn2r100*, and *Slc22a3* expression were significantly upregulated in 40°C–10°C exposure group. Sub-ontology BP analysis in the top DEGs in extreme temperature fluctuations (40°C–10°C) compared to control showed that immune response-regulating and -activating cell surface receptor signaling pathway, activation of immune response, and immune response-activating were significantly upregulated (Figure 2C). KEGG enrichment pathway in extreme temperature fluctuations (40°C–10°C) compared to control showed that Th17 cell differentiation, endocytosis, and T cell receptor signaling pathway were significantly upregulated (Figure 2D). Sub-ontology and KEGG in 40°C and 10°C exposures, compared to the control, are further illustrated in Supplementary Figure 3. Figure 2E illustrates the clustering of airway epithelial cells identified within mice lung, influenced by control (22°C), both extreme temperatures (40°C and 10°C), and temperature fluctuations (40°C–10°C), showing differences in clustering across each exposure group. We further selected the airway epithelial cells and observed that *Cma1*, *Kit*, *Fdx1*, *Elf1a*, *Cdkn2aipnl*, *Htatsf1*, *Mfsd13a*, *Gtf2h5*, *Tiam2*, and *Trmt10c* were significantly upregulated in 40°C exposure (Figure 2F). In addition, we observed that *Sftpc, Gpr171, Sic34a2, Cox14, Lamp3, Luc7l, Nxnl2, Tmub2, Tob1*, and *Cd3e* were significantly upregulated in 10°C exposure, while *Hbb-bs, Arhgef18, Ankrd11, Rnf130, Osbpl5, Vamp8, Tfdp1, Ric8a, Raf1*, and *Rbm25* were significantly upregulated in 40°C–10°C exposure group.

### 3.3 Gene expression in airway epithelial samples from pediatric asthma patients modulated by extreme temperature

**FIGURE 3:**
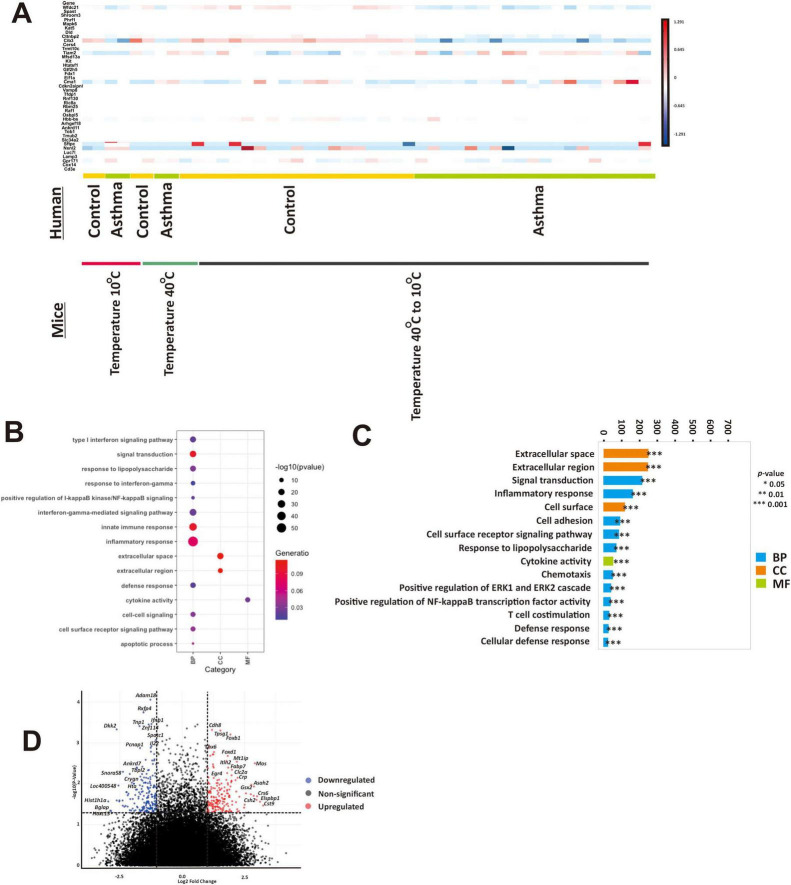
Extreme temperatures play a crucial role in modulating gene expression in airway epithelial cells of pediatric asthma patients. **(A)** A comprehensive comparative analysis of the foremost differential gene expression profiles identified in pediatric asthma subjects in different temperature exposure, alongside the differential gene expression patterns in the control group. The color intensity within these heatmaps represents the activation z-score, with red indicating upregulation and blue signifying downregulation. **(B)** A comprehensive gene ontology enrichment analysis assessed the significantly enriched biological processes (BP), cellular components (CC), and molecular functions (MF) with a focus on the top 15 differentially expressed genes in the extreme temperature fluctuation group. The color depth within this analysis corresponds to the gene ratio, while the point’s size conveys the significance value. **(C)** Top 15 gene ontology enrichment analysis related to biological processes (BP), molecular functions (MF), and cellular components (CC) in pediatric asthma subjects in the extreme temperature fluctuation group. *p < 0.05, **p < 0.01, ***p < 0.001. **(D)** A volcano plot visualize the distribution of differentially expressed genes in pediatric asthma subjects relative to the control group in the extreme temperature fluctuation group. Upregulation is denoted by the color red, while downregulation is indicated by the color blue.

We further investigated the top 10 highly expressed genes from the scRNA-seq analysis of mice in relation to pediatric airway epithelial cell datasets to identify conserved gene regulation that may enhance our understanding of asthma across different age groups (Figure 3A). We observed that the pediatric asthma subjects in the extreme high temperature group exhibited higher expression of *Tiam2* and *Cma1*, whereas *Wfdc21*, *Cib3*, and *Sftpc* expression were decreased. Pediatric asthma subjects in the extreme low temperature group showed decreased expression of *Wfdc21, Cib3, Cma1*, and *Dld*, while *Sftpc* and *Nxnl* expression increased. In addition, asthma subjects in the extreme temperature fluctuation group showed decreased expression of *Wfdc21, Cib3, Gpr171*, and *Cttnbp2*, while *Tiam2* and *Cma1* expression increased. Further sub-ontology BP analysis revealed enrichment in innate immune response, signal transduction, inflammatory response, apoptotic process, cell surface receptor signaling pathway, cell-cell signaling, cytokine activity, interferon-gamma-mediated signaling pathway, positive regulation of I-kappaB kinase/NF-kappa B signaling, type 1 interferon signaling pathway, defense response, and response to interferon gamma (Figures 3B, C). Sub-ontology cellular component analysis showed that extracellular space and extracellular region were most significantly upregulated in asthma subjects. Molecular function analysis showed upregulation of response to lipopolysaccharide in asthma subjects with the temperature fluctuation. In asthma patients in the extreme temperature fluctuations group (40°C–10°C), 594 genes were significantly upregulated whereas 643 were downregulated compared to control (*p* < 0.05) (Figure 3D). Sub-ontology and KEGG in extreme heat and extreme cold in pediatric asthma subjects are shown in Supplementary Figure 4. We observed that innate immune response and inflammatory response were significantly upregulated.

### 3.4 Gene expression in airway epithelial cells associated with adult asthma severity, modulated by extreme temperature

**FIGURE 4:**
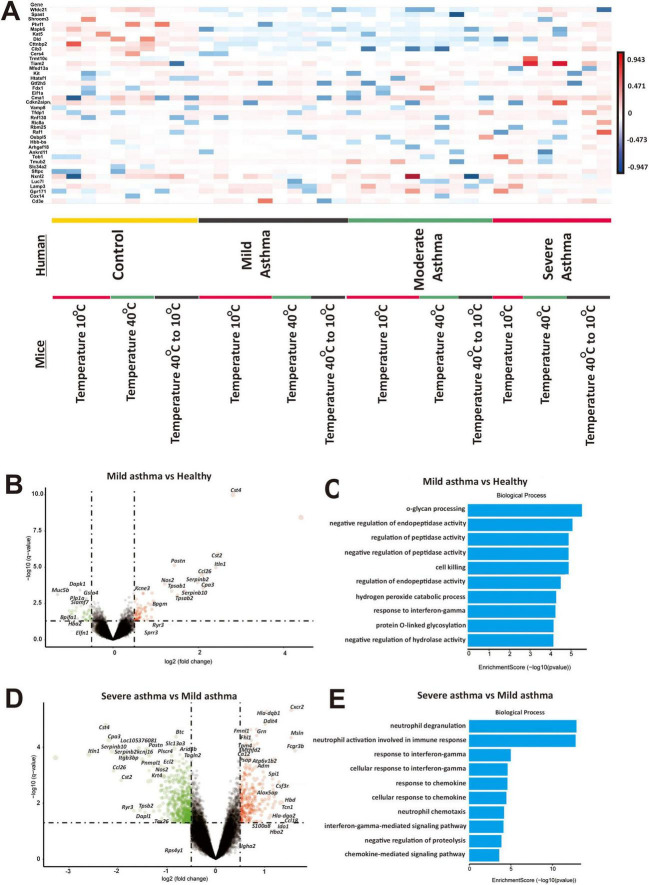
Extreme temperatures play a crucial role in modulating gene expression based on severity in airway epithelial cells of adult asthma patients. **(A)** A comprehensive comparative analysis of the foremost differential gene expression profiles identified in adult human subjects in different temperature exposure, alongside the differential gene expression patterns in the control group. The color intensity within these heatmaps represents the activation z-score, with red indicating upregulation and blue signifying downregulation. **(B)** A volcano plot visualize the distribution of differentially expressed genes in airway epithelial cells in mild asthma subjects relative to the healthy group. Upregulation is denoted by the color red, while downregulation is indicated by the color green. **(C)** A comprehensive gene ontology enrichment analysis assessed the significantly enriched biological processes (BP) with a focus on the top differentially expressed genes in mild asthma subjects relative to the healthy group. **(D)** A volcano plot visualize the distribution of differentially expressed genes in airway epithelial cells in severe asthma subjects relative to the mild asthma subjects. Upregulation is denoted by the color red, while downregulation is indicated by the color green. **(E)** A comprehensive gene ontology enrichment analysis assessed the significantly enriched biological processes (BP) with a focus on the top differentially expressed genes in severe asthma subjects relative to the mild asthma subjects.

We selected the top 10 highly expressed genes in the scRNA-seq analysis from mice to the adult asthma airway epithelial cell datasets to identify conserved gene regulation that may enhance our understanding of asthma across different age groups (Figure 4A). Comparing to the respective control, we observed that the mild, moderate, and severe asthma subject in the extreme low temperature group showed increased *Tob1, Mub2, Sic34a2, Sftpc, Nxnl, Luc71, Lamp3, Gpr171, Cox14*, and *Cd3e* expression, while in the severe asthma subjects showed increased expression in all temperature exposure group. In addition, we observed that mild, moderate, and severe asthma in the extreme low temperature group showed increased expression of *Mfsd13a, Kit, Htatsf1, Gtf2h5, Fdx1, Eif1a*, and *Cma1*. We further observed that mild and moderate asthma subjects in the extreme high, low, and temperature fluctuation groups showed decreased expression of *Wfdc21, Spast, Shroom3, Phrf1, Mapk6, Kat5, Dld, Cttnbp2, Cib3*, *Cma1*, *Sftpc* and *Cers4*, while the severe asthma subjects showed increased expression. We identified 76 upregulated and 37 downregulated genes in mild asthma compared to control (Figure 4B). Sub-ontology biological process (BP) analysis in the top DEGs in mild asthma compared to control showed that o-glycan processing, negative regulation of endopeptidase, and regulation of peptidase activity were significantly upregulated (Figure 4C). We identified 259 upregulated and 604 downregulated genes in severe compared to mild asthma (Figure 4D). In addition, in the top DEGs in severe compared to mild asthma showed neutrophil degranulation, neutrophil activation involved in immune response, and response to interferon gamma were significantly upregulated (Figure 4E). Sub-ontology of BP in moderate asthma subjects compared to severe and mild asthma subjects are shown in Supplementary Figure 5. We observed that regulation of leukocyte mediated immunity and antigen processing and presentation of endogenous antigen were significantly upregulated.

### 3.5 Protein-protein interactions and gene-gene interactions of the top genes in mice and human asthma in regulation with extreme temperatures and temperature fluctuations

**FIGURE 5:**
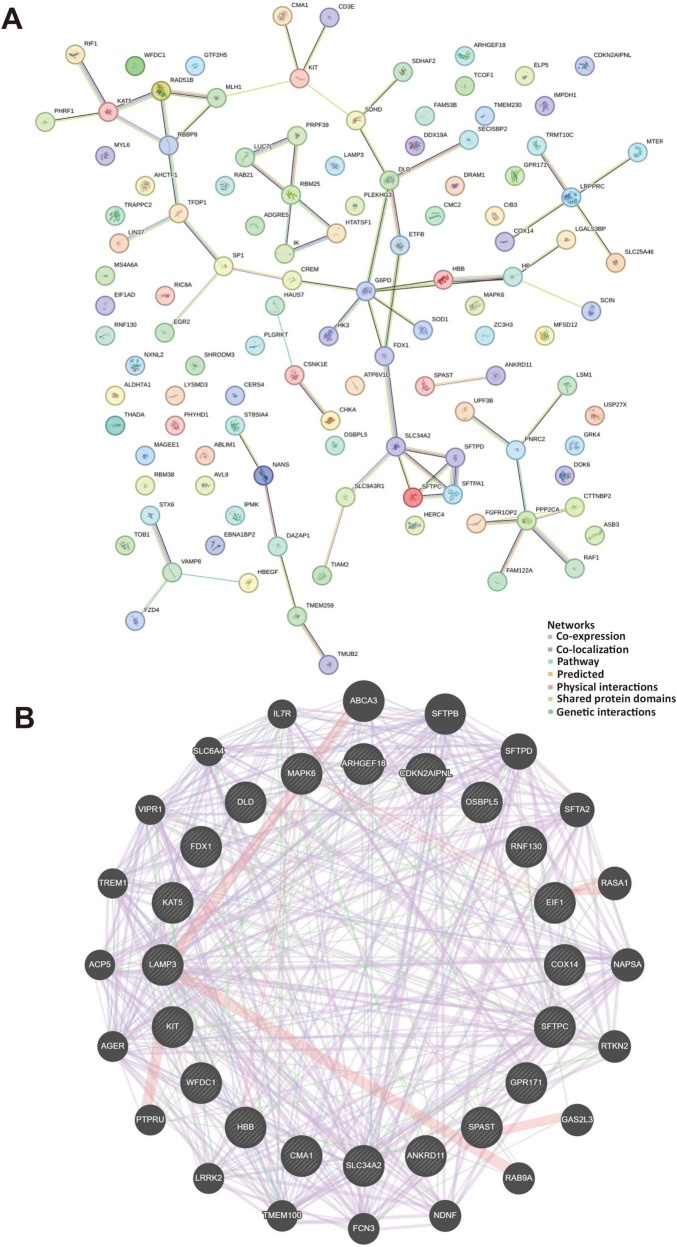
Protein-protein interactions and gene-gene interactions in regulation with extreme temperatures. **(A)** The STRING database contains a network of protein-protein interactions, represented by lines depicting these interactions. The network diagram illustrates 118 gene interactions. **(B)** The gene-gene interaction networks and functions between genes were displayed in various colors. The genes located in the central circle represented the 20 key targets, while those in the outer circle were the genes exhibiting the strongest interactions with these 20 genes. Different colors indicated distinct network modes and functions.

The pivotal targets associated with asthma was investigated and cross-analyzed DEGs observed in mild, moderate and severe asthma in different exposure to temperatures. We observed that the top 10 hub interacted with *Dld, Cma1, Sftpc*, and *Hbb-bs* (Figure 5A). These genes were mainly related to oxidoreductase complex (FDR = 2.88e–13), dihydrolipoyl dehydrogenase complex (FDR = 3.41e–12), tricarboxylic acid cycle enzyme complex (FDR = 1.85e–11), and acyl-CoA metabolic process (FDR = 1.15e–8) (Figure 5B).

## 4 Discussion

The main finding of this study is that we investigated the effects of extreme temperature on the gene expression and pathways from the airway epithelial cells in mouse and asthma patients. The significant findings of this work indicate that extreme high temperature increased *Cma1* and *Tiam2* expression, and extreme low temperature increased *Sftpc* and *Nxnl* expression in asthma, especially among pediatric populations. Further, extreme temperature fluctuations consistently led to decreased expression of *Wfdc21, Cib3, Gpr171*, and *Cttnbp2* in mice and asthma patients. Extreme low temperature decreased *Wfdc21, Cttnbp2, Cib3, and Cma1* expression in mild and moderate asthma, while increased expression in severe asthma. Moreover, extreme temperatures modulated the expression of immune response and lung function genes in the airway epithelium. Additionally, subjects with severe asthma were more sensitive to all extreme temperature conditions. The gene signature of asthma subjects further highlights the role of the airway epithelium in responding to exposure to extreme temperatures in climate change conditions.

Firstly, we observed that *Mmp8*, *Sftpb*, *Cxcl15* and *Cd14* were significantly upregulated in all temperature exposures of airway epithelial cells of our mice model. A previous study in human lung injury patients observed that upregulation in *Mmp8* showed delayed healing process and increased inflammatory responses (18). *Mmp8* contributed to airway hyperresponsiveness and remodeling by altering extracellular matrix turnover, affecting smooth muscle contraction and airway fibroblast invasion (19). Notably, a study emphasized the importance of *Sftpb* in surfactant production, reducing surface tension in the alveoli and preventing collapse during expiration, particularly due to its high expression in epithelial cells compared to endothelial cells and pericytes (20). Several upregulated genes, including *Cxcl15*, were identified and associated with cell cycle, cytoplasmic ribosomal protein, mRNA processing, leukocyte chemotaxis and immune activation (21). A previous study reported that *Cd14* were highly upregulated suggesting a role of immune response and inflammation in immune activation (22). A study also highlighted that warmer temperatures modulate the expression of immune response genes (23). Together, airway epithelial cells were vulnerable to exposure to extreme temperature and temperature fluctuations, which played a role in the regulation of inflammatory responses and immune activation, potentially exacerbating asthma symptoms.

Extreme temperature exposure at 40°C showed significant upregulation of *Cma1* and *Tiam2* expressions in the airway epithelial cells of our mice model. A previous study in heat treatment heifers model observed that several upregulated genes, including *Tiam2*, were identified and associated with cell apoptosis and DNA repair (24). Compared to the human lung tissues with chronic respiratory disease, *Cma1* showed higher expression in bronchial biopsies and lung tissues which induced TNF-α release from macrophages (25). In addition, we observed that *Sftpc* and *Nxnl* expressions were significantly upregulated in temperature 10°C exposure. A previous study that resequenced the *Sftpc* gene in 760 individuals showed that highly upregulation of *Sftpc* were associated with two-fold increased risk for asthma (26). Another study observed that extreme temperatures further regulate *Sftpc* expression, leading to a blockage of cellular macroautophagy (27). Cold temperatures have also been observed to regulate the expression of immune response genes and lung function-related genes in the airway epithelium of individuals with chronic airway diseases (28). In addition, *Nxnl* gene has been linked to stress-induced genome-wide reprogramming and the coordinated transcriptional response to extreme temperatures. We further observed that *Wfdc21, Cib3, Gpr171*, and *Cttnbp2* expression decreased at temperature 40°C to 10°C. A study indicated that *Wfdc21* expression was upregulated in response to extreme temperature changes, potentially aiding in the modulation of inflammatory responses or providing protective effects to tissues (29). In addition, the upregulation of *Cib3* and *Gpr171* indicated the potential roles in stress response and inflammation modulation (30). However, *Cttnbp2* exhibited a variable expression pattern influenced by the duration of temperature exposure (31). We observed that temperature fluctuations activated immune response and promoted lymphocyte differentiation. A previous study observed that temperature fluctuations induced protein misfolding and loss of function, which modulate immune pathways through the hypothalamic-pituitary-adrenal axis (32). These findings suggest that extreme temperatures and temperature fluctuations regulate gene expression in airway epithelial cells.

We further observed that the pediatric asthma subjects in low temperature exposure consistently showed increased *Sftpc* expression compared to our mice model. A previous direct sequencing of the *Sftpc* gene within 101 asthmatic children in Tunisia showed a significant association with asthma disease (33). In an epidemiological study, *Sftpc* was associated with a two-fold increased risk of asthma, but not chronic obstructive pulmonary disease (26). Novel mutations in the *Sftpc* gene (I73T and E66K) were associated with reduced lung function and increased risk of asthma (34). We then observed that the pediatric asthma subjects in high temperature exposure showed increased *Tiam2* and *Cma1* expression. A previous study in children with severe asthma showed that high expression of *Tiam2* was related to regulation of actin cytoskeleton and chemokine signaling pathway (35). A previous study observed that significant association was found between *Cma1* genotypes and total IgE levels in subjects with asthma (36). Mast cell inflammatory mediators play in the allergic immune response, and in particular the role of the mast cell proteases tryptase and chymase in mediating chronic inflammation of the lung in asthma (37, 38). We also observed that more severe asthma was associated with increased neutrophil activation and degranulation in response to extreme temperatures. A previous study identified that neutrophils play a crucial role in driving bronchial inflammation during asthma exacerbations in patients with cold-induced airway conditions (39). Taken together, extreme temperatures and temperature fluctuations play an important role in regulating gene expression in asthmatic patients, especially in pediatric cases.

We observed that extreme low temperature decreased *Wfdc21, Cttnbp2, Cib3*, and *Cma1* expression in mild and moderate asthma, while increased expression in severe asthma. Previous studies have indicated that extreme temperature can influence inflammatory pathways differently across asthma severity level (3, 10). A study observed that *Wfdc21, Cttnbp2. Cib3*, and *Cma1* were significantly upregulated in severe asthma patients (40). Additionally, subjects with severe asthma were more sensitive to low, high temperature and temperature fluctuation conditions, with increased of *Tob1, Mub2, Sic34a2, Sftpc, Nxnl, Luc71, Lamp3, Gpr171, Cox14*, and *Cd3e* expression. A previous study using open database showed that highly expressed genes in severe asthma patients may be related to eosinophilia with notably high Th2 inflammation (41). Previous RNA-seq data showed that inflammation and extracellular matrix remodeling pathways were affected by *Tob1* and *Mub2* expression in several diseases, including asthma (42). In addition, previous study highlighted that *Cib3* high-expression group was mainly enriched in glycerolipid metabolism, folate biosynthesis, cancer-related pathways, and immune response (43). Further, a study observed that *Sftpc, Nxnl, Lamp3, Gpr 171, and Cox14* genes is associated with asthma and the association is even stronger with atopic and severe asthma (44). Additionally, different expressions of the *Cd3e* gene cause various human lung diseases, including asthma, and influence the severity and onset of symptoms (45). Together, severe asthma patients might experience more pronounced physiological changes in response to extreme temperatures.

There are some limitations in our work. The absence of direct clinical data from the asthma dataset limits the direct translation of our findings to real-world scenarios despite our efforts in assessing the comparability of these changes with those observed in asthma. Future studies comparing lung cell types within asthma airway epithelial samples, incorporating vulnerable populations assessments, may be needed to enhance the generalizability of our results. Additionally, further investigations using asthma murine models exposed to extreme temperatures and in vitro experiments with airway epithelial cells inflammatory factors are needed to provide deeper mechanistic insights.

## 5 Conclusion

Our study highlights the gene signature of the airway epithelium, and specifically observed changes in *Cma1* and *Tiam2* expression in extreme high temperature, increased *Sftpc* and *Nxnl* expression in extreme low temperature, and decreased *Wfdc21, Cib3, Gpr171*, and *Cttnbp2* in extreme temperature fluctuation of mice and asthma patients. Extreme temperature decreased *Wfdc21, Cttnbp2, Cib3, and Cma1* expression in mild and moderate asthma, while increased expression in severe asthma patients. Additionally, subjects with severe asthma were more sensitive to all extreme temperature conditions. Extreme temperature effects in the airway epithelium further modulated the activation of the immune response. These findings suggest that extreme temperatures modulate gene expression in the airway epithelium, potentially serving as biomarkers for climate change. Understanding how temperature extremes and fluctuations affect these cells may offer insights into asthma’s pathophysiology and treatment.

## Data Availability

The datasets presented in this study can be found in online repositories. The names of the repository/repositories and accession number(s) can be found in the article/Supplementary material.

## Footnotes

1
https://cran.r-project.org/web/packages/Seurat/index.html

2
https://www.ncbi.nlm.nih.gov/geo/

3
https://www.noaa.gov/

4
https://www.string-db.org/

5
https://genemania.org/
