# Supplementary material for: Extreme temperatures modulate gene expression in the airway epithelium of the lungs in mice and asthma patients
Source: Front Med (Lausanne). 2025 Apr 17;12:1531154. doi: 10.3389/fmed.2025.1531154 (PMC12043461; doi:10.3389/fmed.2025.1531154)
Supplement: Supplementary file 1 [file Table_1.docx]

**Extreme temperatures modulate gene expression in the airway epithelium of the lungs in mice and asthma patients**

Firdian Makrufardi^1,2^ (MD), Syue-Wei Peng^3^ (PhD), Kian Fan Chung^4^ (MD, DSc), Marc Chadeau-Hyam^5,6^ (PhD), Kang-Yun Lee^7,8^ (MD, PhD), Ta-Chih Hsiao^9^ (PhD), Kin-Fai Ho^10^ (PhD), Desy Rusmawatiningtyas^2^ (MD, MSc), Indah Kartika Murni^2^ (MD, PhD), Eggi Arguni^2^ (MD, PhD), Yuan-Hung Wang^11,12^ (PhD), Shu-Chuan Ho^3,8^ (PhD), Feng-Ming Yang^3^ (PhD), Kai-Jen Chuang^13,14^ (PhD), Sheng-Chieh Lin^15,16*^ (MD, PhD), Hsiao-Chi Chuang^3,4,8,17*^ (PhD)

^1^International Ph.D. Program in Medicine, College of Medicine, Taipei Medical University, Taipei, Taiwan

^2^Department of Child Health, Faculty of Medicine, Public Health, and Nursing, Universitas Gadjah Mada – Dr. Sardjito Hospital, Yogyakarta, Indonesia

^3^School of Respiratory Therapy, College of Medicine, Taipei Medical University, Taipei, Taiwan

^4^National Heart and Lung Institute, Imperial College London, London, United Kingdom

^5^Department of Epidemiology and Biostatistics, School of Public Health, Imperial College London, London, United Kingdom

^6^MRC Centre for Environment and Health Imperial College London, London, United Kingdom

^7^Division of Pulmonary Medicine, Department of Internal Medicine, School of Medicine, College of Medicine, Taipei Medical University, Taipei, Taiwan

^8^Division of Pulmonary Medicine, Department of Internal Medicine, Shuang Ho Hospital, Taipei Medical University, New Taipei City, Taiwan

^9^Graduate Institute of Environmental Engineering, National Taiwan University, Taipei, Taiwan

^10^JC School of Public Health and Primary Care, The Chinese University of Hong Kong, Hong Kong, China

^11^Graduate Institute of Clinical Medicine, College of Medicine, Taipei Medical University, Taipei, Taiwan

^12^Department of Medical Research, Shuang Ho Hospital, Taipei Medical University, New Taipei City, Taiwan

^13^School of Public Health, College of Public Health, Taipei Medical University, Taipei, Taiwan

^14^Department of Public Health, School of Medicine, College of Medicine, Taipei Medical University, Taipei, Taiwan

^15^Department of Pediatrics, School of Medicine, College of Medicine, Taipei Medical University, Taipei, Taiwan

^16^Division of Allergy, Asthma, and Immunology, Department of Pediatrics, Shuang Ho Hospital, Taipei Medical University, New Taipei City, Taiwan

^17^Cell Physiology and Molecular Image Research Center, Wan Fang Hospital, Taipei Medical University, Taipei, Taiwan

**Running Title:** Extreme temperatures modulate gene expression in asthma

**Word count:** 3670 [excluding abstract and references]; 5 figures

***Corresponding Author**

*Sheng-Chieh Lin, MD, PhD*

Department of Pediatrics, School of Medicine, College of Medicine, Taipei Medical University, 250 Wuxing Street, Taipei 11031, Taiwan.

Telephone: +886-2-22490088 ext. 2951. Fax: +886-2-22490088. E-mail: [jacklinbox](mailto:r92841005@ntu.edu.tw)@tmu.edu.tw

*Hsiao-Chi Chuang, PhD*

Inhalation Toxicology Research Lab (ITRL), School of Respiratory Therapy, College of Medicine, Taipei Medical University, 250 Wuxing Street, Taipei 11031, Taiwan.

Telephone: +886-2-27361661 ext. 3513. Fax: +886-2-27391143. E-mail: [chuanghc@tmu.edu.tw](mailto:r92841005@ntu.edu.tw)

**Materials and Methods**

***Thermal exposure system***

We have developed an advanced thermal whole-body exposure system for rodents, modified from our previous works [1-3]. The system features an inlet air stream that went through HEPA filters to remove particulate matter and charcoal or denuder to eliminate gaseous pollutants and organic compounds. Mice were subjected to different temperatures at consistent relative humidity levels (65%) within the system, which was outfitted with thermal meters and humidity monitors for accurate control and monitoring. In this study, the B6.*Sftpc-CreER^T2^;Ai14(RCL-tdT)*-D mice in both gender were exposed to normal (22°C), extreme low (10°C), high temperature (40°C) and temperature fluctuation (40°C for 2 hrs then 10°C for next 2 hrs) . The exposure was 4 hrs/day for 7 days.

***Single cell RNA sequencing data***

Whole lung sample was collected from each mouse in normal (22°C), extreme low (10°C), high temperature (40°C) and temperature fluctuation (40°C to 10°C), and the lung was perfused to eliminate red blood cells. Lung dissociation was performed. A gentleMACS C Tube was added to 2.4 ml of 1x buffer saline, 100µL of enzyme D, and 15 µL of enzyme A. The whole lung sample was dissected into single lobes. The lobes were transferred into the gentleMACS C tube containing the enzyme mix. The C tube was tightly closed and attached upside down onto the sleeve of the gentleMACS Dissociator. Program 37C_m_LDK_1 was performed using the heating function of the gentleMACS Octo Dissociator. After termination of the program, the C Tube was detached from the gentleMACS Dissociator. A short centrifugation (1500 rpm, 5 min) was performed to collect the sample material at the tube bottom. A 1mL of PEB buffer was added to resuspend the sample and apply the cell suspension to a cell strainer (70 µm) placed on a 50 mL tube. The cell strainer (70 µm) was washed with 1 mL of PEB buffer. The cell strainer (70 µm) was discarded, and the cell suspension was centrifuged at 1500 rpm for 3 min. The supernatant was completely aspirated. 1-3mL of 1x RBC lysis was added to lyse red blood cells in single-cell suspension. A short centrifugation step (1500 rpm, 3 min) was performed, and the supernatant was aspirated completely. 200µL of dead cell removal beads were added and incubated for 15 minutes at room temperature. Then, 500µL of 1x binding buffer was added. Magnetic cell separation was performed followed by a positive selection protocol. The cell suspension was centrifuged at 1500 rpm for 10 min. The supernatant was completely aspirated, and 1mL of 1×PBS buffer was added to resuspend the sample. The cell count and viability were checked, and cell pellets were collected. Cell pellets were analyzed using the GemCode Single Cell Platform using the Chromium Fixed RNA Kit (10X Genomics, Pleasanton) following the protocol provided by the company. In brief, cell suspensions were fixed with paraformaldehyde. After probe hybridization, cells were pooled and loaded onto a Chromium Single-Cell Chip Q along with the reverse transcription (RT) master mix and single cell TL gel beads, aiming for (Target cell number) cells per channel. Following generation of single-cell gel bead-in-emulsions (GEMs), reverse transcription was performed using a Applied Biosystems™ Veriti™ 96-Well Thermal Cycler (Thermo Fisher Scientific, USA). Amplified cDNA was purified using SPRIselect beads and converted to the sequencing library according to the manufacturer’s protocol. Library was sequenced on an Illumina NovaSeq platform following read length: 150 bp Read 1 (16bp single cell barcode, 10x barcode; 12bp Unique molecular identifier, UMI), 150 bp Read 2 (transcript insert), 10 bp i7 Index (sample index), 10 bp i5 Index (sample index). To analyze the scRNA-Seq information, the Cell Ranger software pipeline (version 2.0) provided by 10X Genomics was employed. Briefly, the data was de-barcoded, and the nucleotide reads were mapped to the genome and transcriptome with the Spliced Transcripts Alignment to a Reference (STAR) aligner software (CSC, Espoo, Finland). The R package Seurat (version 1.4.0.14; https://cran.r-project.org/web/ packages/Seurat/index.html) was used for processing the UMI count matrix and removal of potential multiple captures [4]. Normalized aggregate data across samples was generated to produce a matrix of gene counts versus cells. Cell prefiltering was conducted based on quality control standards, encompassing the expression of 500–1800 genes, with mitochondrial gene mapping constituting less than 20% of UMIs, and the total UMIs amounting to less than 20,000. The process of automatically labeling cell types within clusters derived from single-cell RNA sequencing data was executed utilizing scCATCH [5]. Loupe™ Cell Browser v2.1 (10X Genomics) was used to perform analysis in gene expressions in epithelial cells.

***Gene expression datasets***

We selected gene expression datasets from the Gene Expression Omnibus (GEO), a publicly accessible database managed by the National Center for Biotechnology Information (NCBI). The GEO database (https://www.ncbi.nlm.nih.gov/geo/) serves as a comprehensive repository for high-throughput gene expression data. Our selection focused on datasets relevant to our research objectives, which aim to explore patterns of gene expression across various biological conditions and experimental setups. The dataset criteria were established as follows: (a) the dataset must include both healthy controls and asthmatic patients, and (b) the samples must be derived from airway epithelium. Transcriptomics data from samples of airway epithelium brushings were included in the analysis. Gene expression data were gathered from 23 healthy controls and 23 patients with asthma in pediatric populations. Additionally, data from epithelial brushings were collected from a diverse group, including 10 healthy controls, 10 patients with mild asthma, 10 patients with moderate asthma, and 8 patients with severe asthma among adult participants.

**Table S1.** List of publicly available airway epithelial samples processed asthma data sets used

| **Populations** | **Reference** |
| --- | --- |
| Pediatric asthma | Wesolowska-Andersen, et al [6], Del Duca, et al [7] |
| Adult asthma | Yan, et al [8] |

**Table S2.** The characteristics and clinical attributes of patients enrolled in the nasal and bronchial airway epithelial gene analysis.

| **Variables** | **Total** | **Control group** | **Extreme low temperature** | **Extreme high temperature** | **Extreme temperature fluctuation** |
| --- | --- | --- | --- | --- | --- |
| **Gender** |  |  |  |  |  |
| Male, n (%) | 19 (50.0) | 4 (40.0) | 9 (75.0) | 3 (33.3) | 3 (42.9) |
| Female, n (%) | 19 (50.0) | 6 (60.0) | 3 (25.0) | 6 (66.7) | 4 (57.1) |
| Asthma control questionnaire, mean±SD | 1.84±1.59 | 2.18±1.14 | 1.06±0.64 | 2.68±2.60 | 1.61±1.14 |
| **Smoking status** | |  |  |  |  |
| Current smoker, n (%) | 6 (15.8) | 3 (30.0) | 0 (0.0) | 2 (22.2) | 1 (14.3) |
| Ex-smoker, n (%) | 1 (2.6) | 1 (10.0) | 0 (0.0) | 0 (0.0) | 0 (0.0) |
| Non-smoker, n (%) | 31 (81.6) | 6 (60.0) | 12 (100.0) | 7 (77.8) | 6 (85.7) |
| **Asthma severity** | |  |  |  |  |
| Mild, n (%) | 14 (36.9) | 4 (40.0) | 5 (41.7) | 3 (33.3) | 2 (28.6) |
| Moderate, n (%) | 13 (34.2) | 3 (30.0) | 5 (41.7) | 3 (33.3) | 2 (28.6) |
| Severe, n (%) | 11 (28.9) | 3 (30.0) | 2 (16.7) | 3 (33.3) | 3 (42.9) |
| Allergic rhinitis, n (%) | 23 (60.5) | 3 (30.0) | 9 (75.0) | 5 (55.6) | 6 (85.7) |
| Nasal polyp, n (%) | 5 (13.2) | 3 (30.0) | 1 (8.3) | 1 (11.1) | 0 (0.0) |
| Inhaled corticosteroids, n (%) | 27 (71.1) | 6 (60.0) | 9 (75.0) | 7 (77.8) | 5 (71.4) |
| Oral corticosteroids, n (%) | 3 (7.9) | 1 (10.0) | 0 (0.0) | 1 (11.1) | 1 (14.3) |
| FEV_1_ (L), mean±SD | 2.95±0.98 | 2.68±1.06 | 3.09±0.93 | 3.18±1.18 | 2.80±0.74 |
| FVC (L), mean±SD | 4.05±1.14 | 3.80±1.25 | 3.98±0.98 | 4.34±1.42 | 4.17±0.98 |
| FEV_1_/FVC (%), mean±SD | 17.73±16.49 | 70.01±13.03 | 76.64±9.48 | 70.09±10.38 | 67.43±13.59 |
| Reversibility (%),mean±SD | 71.65±11.58 | 17.10±7.41 | 14.72±6.94 | 27.87±29.67 | 10.78±10.14 |
| **GINA treatment control classification** | | |  |  |  |
| 1 | 8 (21.1) | 1 (10.0) | 3 (25.0) | 2 (22.2) | 2 (28.6) |
| 2 | 13 (34.2) | 2 (20.0) | 7 (58.3) | 3 (33.3) | 1 (14.3) |
| 3 | 17 (44.7) | 7 (70.0) | 2 (16.7) | 4 (44.4) | 4 (57.1) |


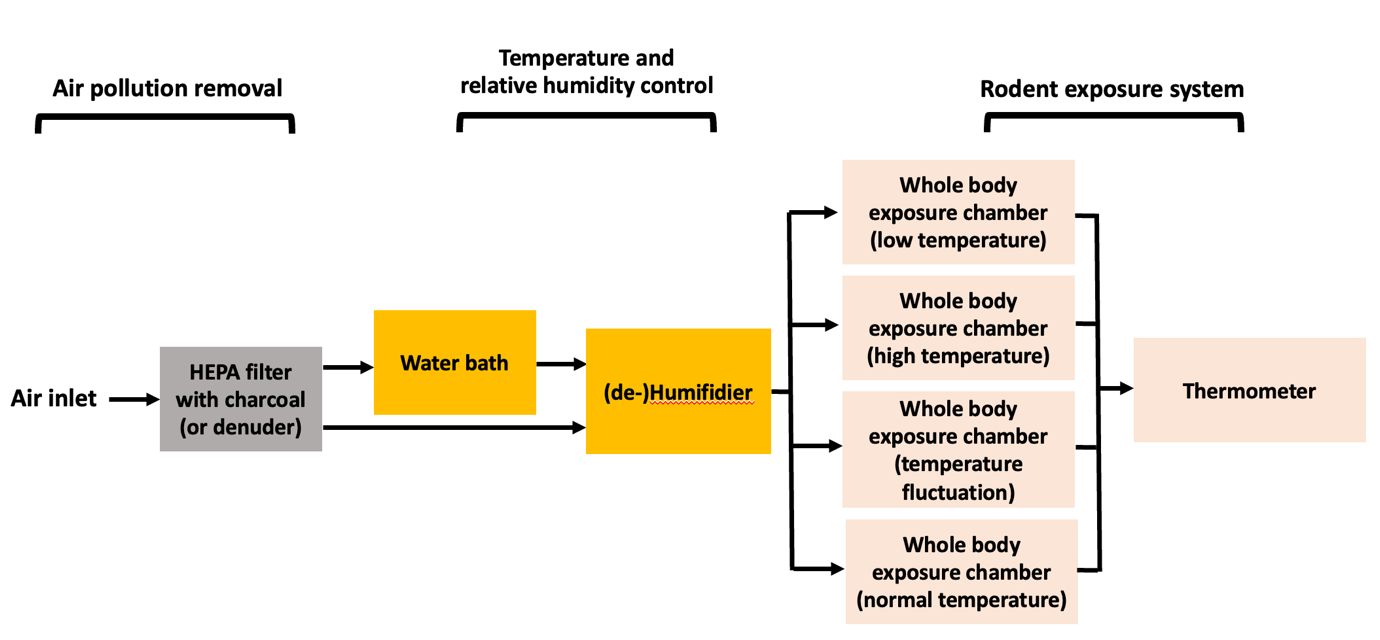


**Figure S1.** The illustration of the thermal whole-body exposure system in mice


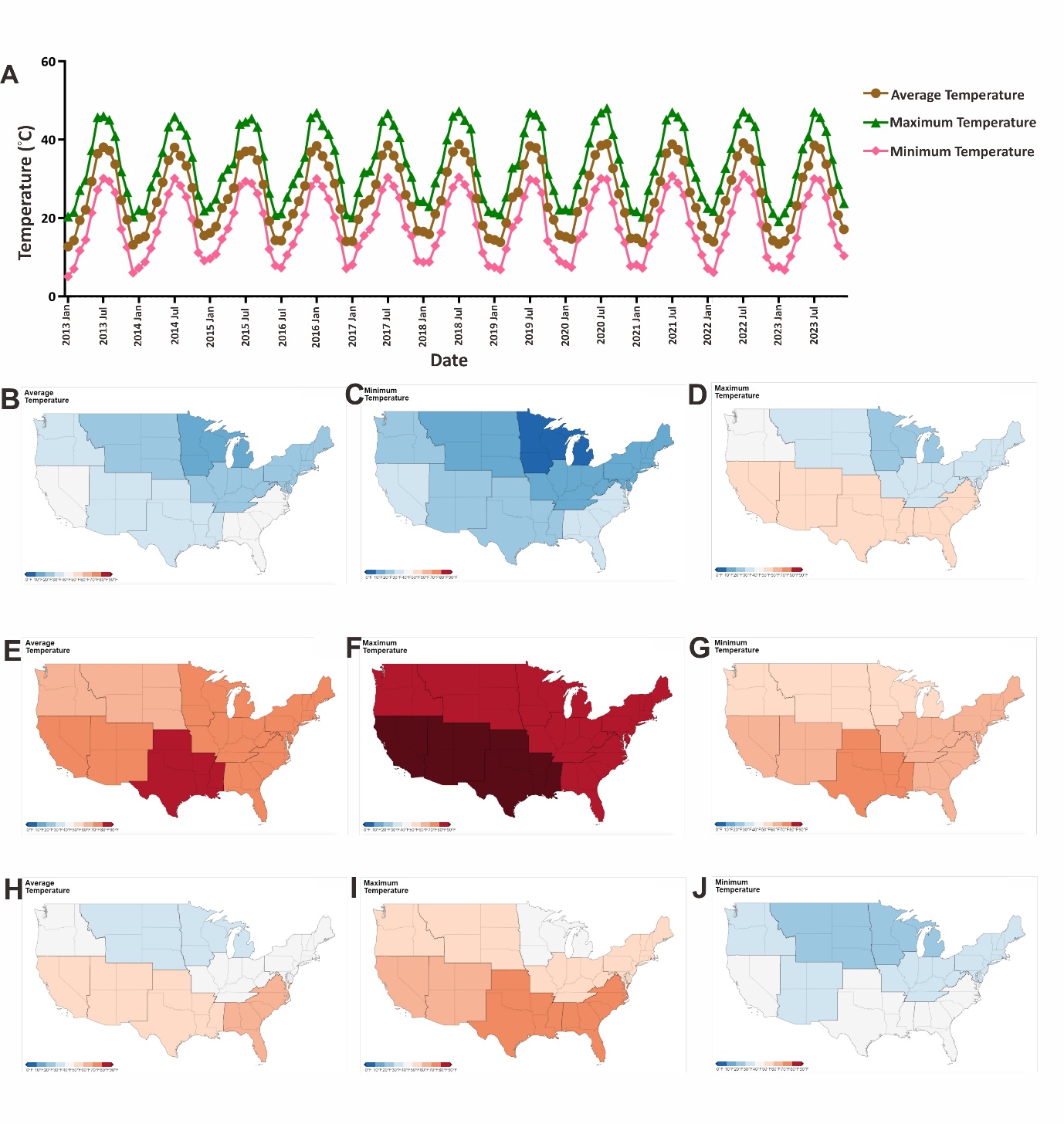


**Figure S2. RNA sequencing analysis of human samples across various states within the United States reveals distinct temperature-dependent gene expression profiles**

A. The study period witnessed fluctuations in temperature over the course of the month, highlighting dynamic climatic variations

B. The average temperatures varied among states, reflecting geographical differences in the populations during the winter season

C. The minimum temperatures varied among states, reflecting geographical differences in the populations during the winter season

D. The maximum temperatures varied among states, reflecting geographical differences in the populations during the winter season

E. The average temperatures varied among states, reflecting geographical differences in the populations during the summer season

F. The minimum temperatures varied among states, reflecting geographical differences in the populations during the summer season

G. The maximum temperatures varied among states, reflecting geographical differences in the populations during the summer season

H. The average temperatures varied among states, reflecting geographical differences in the populations during the spring/fall season

I. The minimum temperatures varied among states, reflecting geographical differences in the populations during the spring/fall season

J. The maximum temperatures varied among states, reflecting geographical differences in the populations during the spring/fall season

**
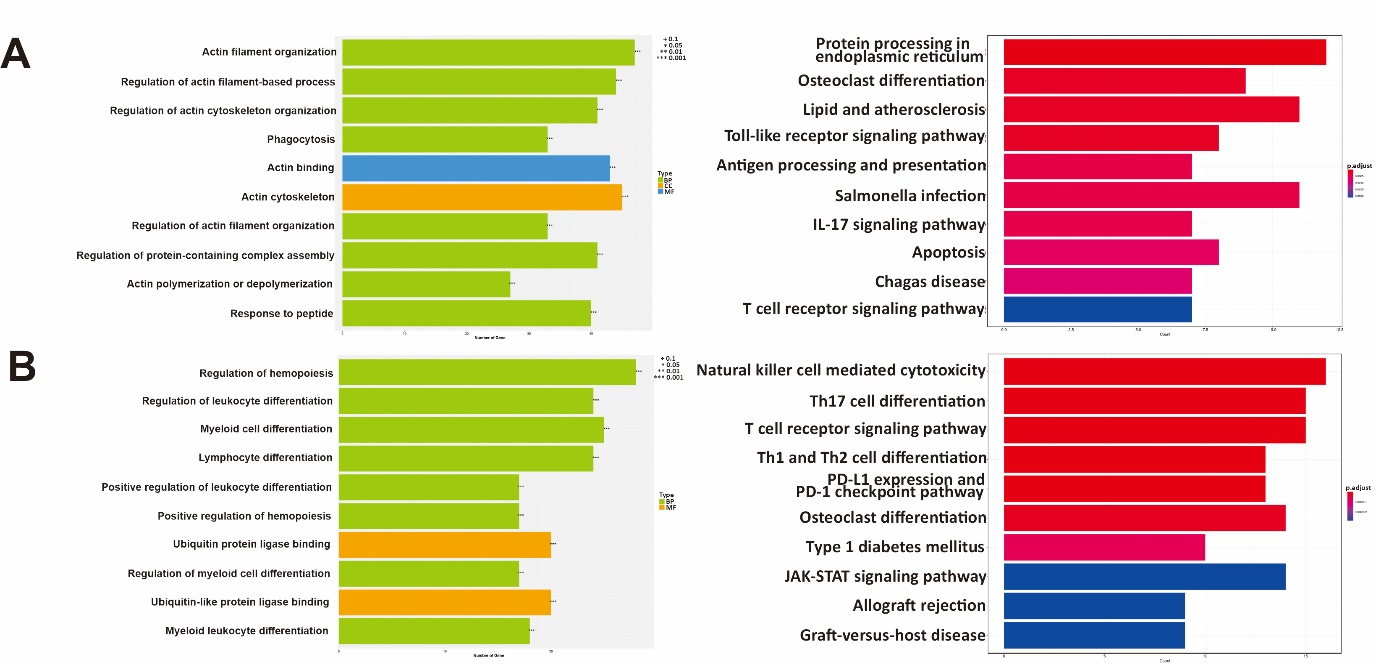
**

**Figure S3.** **Gene ontology enrichment analysis related to significantly enriched biological processes (BP), molecular functions (MF), and cellular components (CC) with Kyoto Encyclopedia of Genes and Genomes (KEGG) enrichment pathway**

A. Extreme heat (temperature 40°C) compared to control.

B. Extreme cold (temperature 10°C) compared to control.


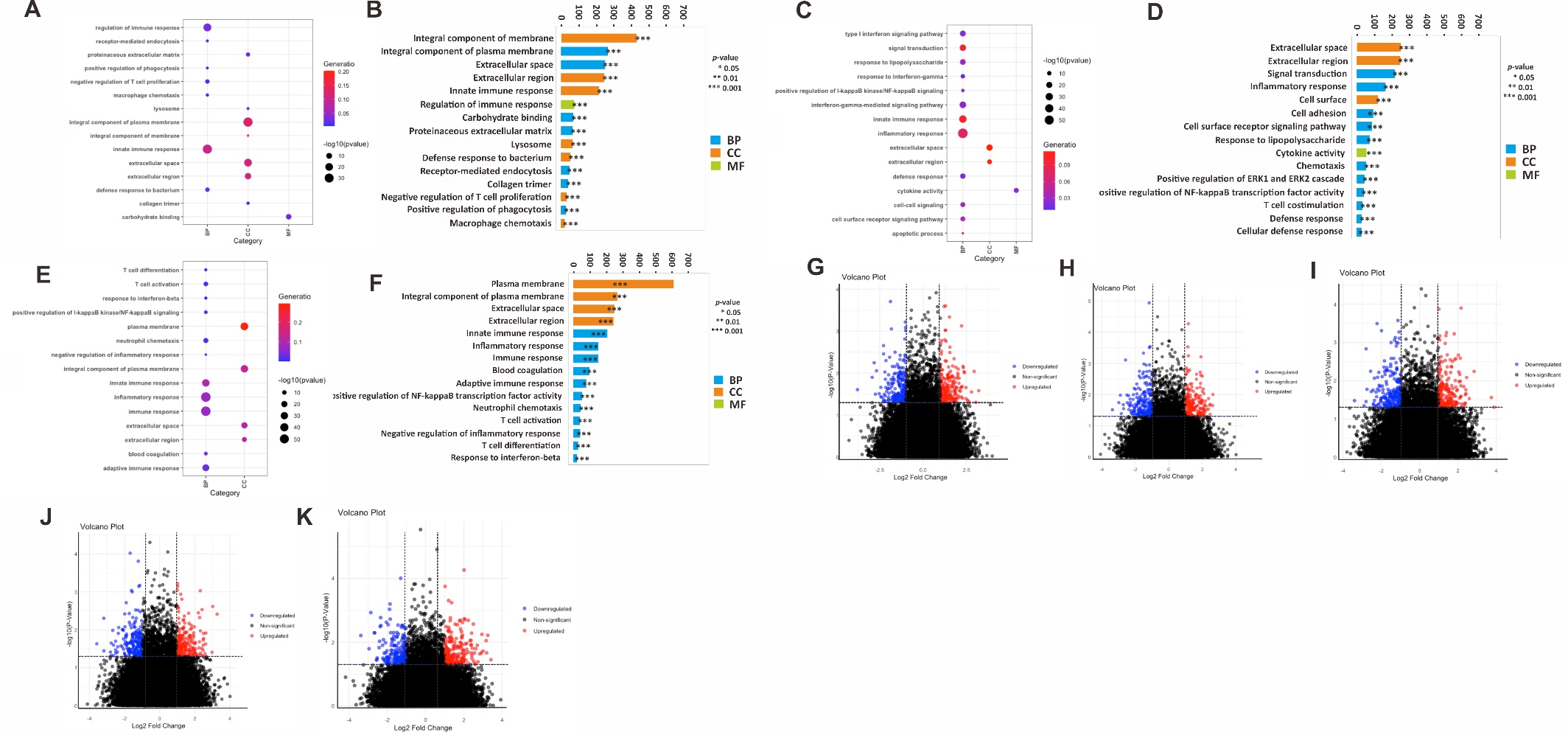


**Figure S4. Extreme temperatures and temperature variations play a crucial role in modulating gene expression in airway epithelial cells of pediatric asthma patients**

**A.** A comprehensive gene ontology enrichment analysis assessed the significantly enriched biological processes (BP), cellular components (CC), and molecular functions (MF) with a focus on the top 15 differentially expressed genes in the 10 degree temperature exposure. The color depth within this analysis corresponds to the gene ratio, while the point's size conveys the significance value.

**B.** Top 15 gene ontology enrichment analysis related to biological processes (BP), molecular functions (MF), and cellular components (CC) in pediatric asthma subjects in the 10 degree temperature exposure. *p<0.05, **p<0.01, ***p<0.001

**C.** A comprehensive gene ontology enrichment analysis assessed the significantly enriched biological processes (BP), cellular components (CC), and molecular functions (MF) with a focus on the top 15 differentially expressed genes in the 40 degree temperature exposure. The color depth within this analysis corresponds to the gene ratio, while the point's size conveys the significance value.

**D.** Top 15 gene ontology enrichment analysis related to biological processes (BP), molecular functions (MF), and cellular components (CC) in pediatric asthma subjects in the 40 degree temperature exposure. *p<0.05, **p<0.01, ***p<0.001

**E.** A comprehensive gene ontology enrichment analysis assessed the significantly enriched biological processes (BP), cellular components (CC), and molecular functions (MF) with a focus on the top 15 differentially expressed genes in the control group. The color depth within this analysis corresponds to the gene ratio, while the point's size conveys the significance value.

**F.** Top 15 gene ontology enrichment analysis related to biological processes (BP), molecular functions (MF), and cellular components (CC) in pediatric asthma subjects in the control group. *p<0.05, **p<0.01, ***p<0.001

**G.** A volcano plot visualize the distribution of differentially expressed genes in pediatric asthma subjects in the 10 degree temperature exposure. Upregulation is denoted by the color red, while downregulation is indicated by the color blue.

**H.** A volcano plot visualize the distribution of differentially expressed genes in pediatric control subjects in the 10 degree temperature exposure. Upregulation is denoted by the color red, while downregulation is indicated by the color blue.

**I.** A volcano plot visualize the distribution of differentially expressed genes in pediatric asthma subjects in the 40 degree temperature exposure. Upregulation is denoted by the color red, while downregulation is indicated by the color blue.

**J.** A volcano plot visualize the distribution of differentially expressed genes in pediatric control subjects in the 40 degree temperature exposure. Upregulation is denoted by the color red, while downregulation is indicated by the color blue.

**K.** A volcano plot visualize the distribution of differentially expressed genes in pediatric control subjects in the 40 to 10 degree temperature exposure. Upregulation is denoted by the color red, while downregulation is indicated by the color blue.


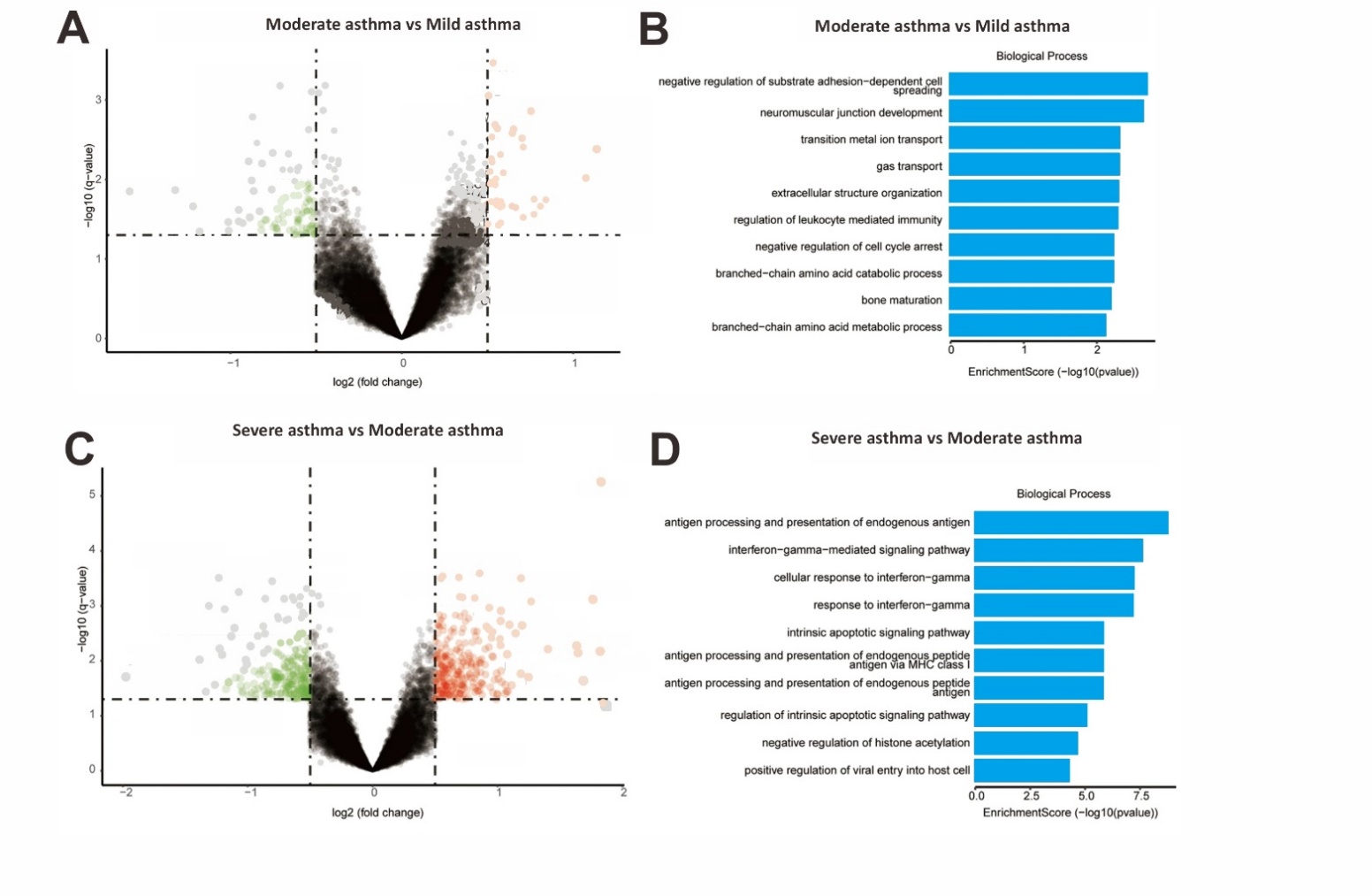


**Figure S5. Volcano plot and gene ontology enrichment analysis in airway epithelial cells of adult asthma patients**

1. A volcano plot visualize the distribution of differentially expressed genes in bronchial airway epithelial cells in moderate asthma subjects relative to mild asthma subjects. Upregulation is denoted by the color red, while downregulation is indicated by the color green.
2. A comprehensive gene ontology enrichment analysis assessed the significantly enriched biological processes (BP) with a focus on the top differentially expressed genes in moderate asthma subjects relative to mild asthma subjects.
3. A volcano plot visualize the distribution of differentially expressed genes in bronchial airway epithelial cells in severe asthma subjects relative to the moderate asthma subjects. Upregulation is denoted by the color red, while downregulation is indicated by the color green.
4. A comprehensive gene ontology enrichment analysis assessed the significantly enriched biological processes (BP) with a focus on the top differentially expressed genes in severe asthma subjects relative to the moderate asthma subjects.

**References**

1. Hsiao T-C, Chang J, Wang J-Y, Wu D, Chuang K-J, Chen J-K, Cheng T-J, Chuang H-C. Serum Neurofilament Light Polypeptide is a Biomarker for Inflammation in Cerebrospinal Fluid Caused by Fine Particulate Matter. *Aerosol and Air Quality Research* 2020.

2. Chuang HC, Chen YY, Hsiao TC, Chou HC, Kuo HP, Feng PH, Ho SC, Chen JK, Chuang KJ, Lee KY. Alteration in angiotensin-converting enzyme 2 by PM(1) during the development of emphysema in rats. *ERJ open research* 2020: 6(4).

3. Shih C-H, Chen J-K, Kuo L-W, Cho K-H, Hsiao T-C, Lin Z-W, Lin Y-S, Kang J-H, Lo Y-C, Chuang K-J, Cheng T-J, Chuang H-C. Chronic pulmonary exposure to traffic-related fine particulate matter causes brain impairment in adult rats. *Particle and Fibre Toxicology* 2018: 15(1): 44.

4. Stuart T, Butler A, Hoffman P, Hafemeister C, Papalexi E, Mauck WM, 3rd, Hao Y, Stoeckius M, Smibert P, Satija R. Comprehensive Integration of Single-Cell Data. *Cell* 2019: 177(7): 1888-1902.e1821.

5. Shao X, Liao J, Lu X, Xue R, Ai N, Fan X. scCATCH: Automatic Annotation on Cell Types of Clusters from Single-Cell RNA Sequencing Data. *iScience* 2020: 23(3): 100882.

6. Wesolowska-Andersen A, Everman JL, Davidson R, Rios C, Herrin R, Eng C, Janssen WJ, Liu AH, Oh SS, Kumar R, Fingerlin TE, Rodriguez-Santana J, Burchard EG, Seibold MA. Dual RNA-seq reveals viral infections in asthmatic children without respiratory illness which are associated with changes in the airway transcriptome. *Genome biology* 2017: 18(1): 12.

7. Del Duca E DD, Kim M, Bar J, Correa Da Rosa J, Rabinowitz G, Facheris P, Gómez Arias PJ, Chang A, Utti V, Chowdhury A, Liu Y, Laculiceanu A, Agache I, Guttman-Yassky E. Transcriptomic evaluation of skin tape-strips in children with allergic asthma uncovers epidermal barrier dysfunction and asthma-associated biomarkers abnormalities. *Allergy* 2024: 79(6): 1516-1530.

8. Yan Q. Transcriptomic analysis of human bronchial epithelium reveals unique immune landscape in severe asthma. *In:* ResearchData, ed., 2022.
